# Plant-Based Diets and Thyroid Homeostasis in Children and Adolescents: Balancing Anti-Inflammatory Potential and Micronutrient Vulnerability

**DOI:** 10.3390/nu18213686

**Published:** 2026-11-06

**Authors:** Giovanni Luppino, Francesca Franchina, Matteo Pontone, Malgorzata Wasniewska, Stefano Stagi

**Affiliations:** 1 Auxoendocrinology Center, Anna Meyer Children’s Hospital, 50139 Florence, Italy; francesca.franchina8@gmail.com (F.F.); matteo.pontone@unifi.it (M.P.); 2 Department of Human Pathology in Adulthood and Childhood “Gaetano Barresi”, University of Messina, 98124 Messina, Italy; malgorzata.wasniewska@unime.it; 3 Department of Health Sciences, University of Florence, 50139 Florence, Italy

**Keywords:** vegan diet, vegetarian diet, thyroid function, childhood, micronutrients

## Abstract

Plant-based diets are increasingly common among children and adolescents because of their potential health benefits, environmental sustainability, and ethical considerations. However, their implications for thyroid health during growth and development remain incompletely characterised. The effects of plant-based diets on thyroid function may depend on overall diet quality and the extent of exclusion of animal-derived foods. Well-planned plant-based diets provide bioactive compounds, dietary fibre, and unsaturated fatty acids that may reduce oxidative stress and inflammation, promote a healthy gut microbiota, and support immune homeostasis. Conversely, poorly planned diets, particularly those rich in ultra-processed foods, may promote inflammation, oxidative stress, and intestinal dysbiosis. Vegetarian and vegan diet patterns may also increase the risk of inadequate intake of iodine, iron, zinc, selenium, and vitamins, while some plant-derived foods, including soy products and cruciferous vegetables, may influence thyroid physiology. These issues are particularly relevant during childhood, when thyroid hormones are essential for neurodevelopment, growth, and metabolic regulation. Current evidence suggests that thyroid health depends primarily on overall diet quality and micronutrient adequacy rather than the exclusion of animal-derived foods itself. This review critically examines these relationships, with particular emphasis on paediatric evidence.

## 1. Introduction

Plant-based diets are becoming increasingly common during childhood and adolescence, reflecting growing interest in environmental sustainability, animal welfare, and potential health benefits [1]. In the United Kingdom, the rate of individuals identifying as vegetarian or vegan increased from approximately 2% in 2010 to 4.5% in 2019, and about 8% of children considered themselves vegan and 13% vegetarian [2]. In Italy, nearly 9% of infants are reported to follow vegetarian or vegan complementary feeding practices [2,3]. This trend poses new challenges for paediatric endocrinologists, who must balance the potential health benefits of plant-based diets with the nutritional requirements of growth and development.

Plant-based diets encompass a spectrum of dietary patterns that differ in the degree of exclusion of animal-source foods, ranging from Mediterranean and pescatarian diets to lacto-ovo-vegetarian, vegetarian, and vegan diets [4,5]. Not all plant-based diets are nutritionally equivalent: their health effects depend on the extent to which animal foods are excluded, overall dietary quality and the degree of food processing. The growing use of ultra-processed vegan alternatives raises concerns because their nutritional profiles may not be equivalent to the foods they replace [6,7].

Well-planned plant-based diets have been associated with lower systemic inflammatory markers and improved cardiometabolic profiles, although evidence regarding immune modulation and thyroid autoimmunity remains limited, particularly in children [8]. Although appropriately planned vegetarian and vegan diets can meet nutritional requirements, they require careful dietary planning and, in some cases, appropriate supplementation to prevent deficiencies in key micronutrients. Specific micronutrients such as iodine, selenium, iron, zinc, and some vitamins are generally present in low concentrations in plant-based foods, and their absorption may be inhibited by factors found in vegan and vegetarian diets.

The thyroid axis represents a system of specific interest, as the synthesis, peripheral conversion, and action of triiodothyronine (T3) and thyroxine (T4) require an adequate and coordinated supply of micronutrients. Furthermore, inflammatory pathways and alterations in oxidative status may influence thyroid function and immune homeostasis [2,4]. During the paediatric age, thyroid function is involved in several systemic functions, including foetal and neonatal brain development, linear growth during childhood and the pubertal stage, and the regulation of basal metabolism. Childhood represents a biologically unique period because rapid growth, neurodevelopment, and maturation of endocrine and immune systems may amplify the consequences of even mild nutritional inadequacies. Poorly planned restrictive plant-based diets in children may compromise nutritional adequacy; whether this might lead to thyroid dysfunction in children remains uncertain [9,10]. Most available evidence derives from adult populations, while paediatric studies are scarce and frequently focus on nutritional status rather than thyroid outcomes. This review examines current evidence regarding the relationship between plant-based diets and thyroid health in children, with particular emphasis on dietary quality, micronutrient adequacy, inflammatory mechanisms and thyroid autoimmunity.

## 2. Materials and Methods

A targeted literature search was conducted in PubMed/MEDLINE using combinations of terms related to plant-based dietary patterns, including “plant-based”, “vegetarian”, “vegan”, and “ultra-processed foods”, together with terms related to thyroid health, micronutrient status, thyroid diseases and autoimmunity, oxidative stress, inflammation, and the gut microbiota. Paediatric search terms included “infant”, “child”, “adolescent”, “paediatric”, “puberty”, and “neurodevelopment”. Priority was given to paediatric studies, systematic reviews, meta-analyses, clinical guidelines, consensus statements, and position papers. Relevant adult and experimental studies were considered only when paediatric evidence was unavailable or when they contributed to the interpretation of biological mechanisms. Additional publications and authoritative nutritional recommendations were identified through manual examination of the reference lists of key articles. The evidence was organised according to the principal thematic areas addressed in this review. Given the limited availability of direct paediatric evidence on plant-based diets and thyroid health and the heterogeneity of the available studies, a narrative approach was adopted to integrate clinical findings with relevant mechanistic evidence. This review was not intended as a systematic assessment of the literature; therefore, a PRISMA flow diagram and a formal risk-of-bias assessment were not included.

## 3. Plant-Based Diets, Biological Mechanisms, and Thyroid Homeostasis

Dietary patterns are important environmental determinants of thyroid function, influencing nutrient availability, cellular metabolism, inflammatory, immune responses, and gut microbiota composition. Their role is particularly relevant during childhood, when thyroid hormones are essential for growth, skeletal maturation, and neurocognitive development. Overall dietary quality may help maintain thyroid homeostasis during periods of increased nutritional and metabolic demand [2,9].

Diets characterised by a high intake of plant foods are generally associated with a more favourable metabolic and inflammatory profile than Western diets, which are often rich in saturated fats, refined sugars, and ultra-processed foods (UPFs). Diets based on minimally processed plant foods provide fibre, polyphenols, carotenoids, and other bioactive compounds that may contribute to the regulation of oxidative stress and inflammatory pathways [11,12]. Among predominantly plant-based diets, the Mediterranean diet (MD) has been the most extensively investigated. Although not exclusively plant-based, it is characterised by a high intake of fruits, vegetables, legumes, whole grains, nuts, and extra virgin olive oil, together with moderate consumption of animal-derived foods. The diet has been associated with anti-inflammatory, antioxidant, and immunomodulatory effects through modulation of metabolism, gut microbiota composition, and cellular redox balance [9,13].

The thyroid gland is especially susceptible to oxidative stress because thyroid hormone synthesis physiologically requires the generation of reactive oxygen species (ROS). Under physiological conditions, ROS generation is tightly controlled by endogenous antioxidant systems. Excessive ROS production or impaired antioxidant capacity may promote cellular damage and inflammatory signalling, thereby contributing to the pathogenesis of autoimmune thyroid disease [14,15]. Plant-derived bioactive compounds, including polyphenols, flavonoids, carotenoids, and antioxidant vitamins, may contribute to the regulation of oxidative stress and inflammatory responses by modulating signalling pathways such as nuclear factor kappa-light-chain-enhancer of activated B cells (NF-κB) and mitogen-activated protein kinase (MAPK), which regulate inflammatory signalling and cellular stress responses [9,16]. Omega-3 polyunsaturated fatty acids (ω-3 PUFAs), derived from α-linolenic acid in some plant foods and provided in higher amounts by diets including fish, also participate in inflammatory regulation through the production of specialised pro-resolving mediators, thereby contributing to immune homeostasis and reduced pro-inflammatory signalling [17,18]. The gut–thyroid axis may provide an additional indirect link between diet and thyroid homeostasis through modulation of the gut microbiota, intestinal barrier, and immune function [17,18]. These proposed pathways are supported largely by adult and experimental evidence; their clinical relevance to children following plant-based diets remains uncertain.

### 3.1. Dietary Quality and Food Processing in Plant-Based Diets

The health effects of plant-based diets depend not only on the proportion of plant-derived foods but also on their overall quality and degree of processing. The classification of a diet as “plant-based” alone does not adequately define its biological properties [19].

A diet primarily based on whole or minimally processed plant foods, including legumes, whole grains, fruits, vegetables, seeds, and nuts, differs from one characterised by a high intake of UPFs, which are generally associated with lower nutritional density and reduced content of bioactive compounds [20,21].

According to the NOVA classification, UPFs are industrial formulations characterised by extensive processing and modifications of the original food matrix. This distinction is particularly relevant in plant-based diets, as highly processed plant-based substitutes may differ substantially in nutritional quality from minimally processed plant foods [20,22].

High consumption of UPFs has been associated with lower overall dietary quality and increased risk of obesity, insulin resistance, and cardiometabolic alterations, including during childhood. These associations may be related not only to higher energy density and lower fibre and micronutrient content, but also to food additives, alterations in food structure, and potential effects on gut microbiota composition [23,24,25,26].

Overall, dietary quality and food processing may represent important determinants of thyroid health beyond the simple distinction between plant- and animal-derived foods. Although direct evidence linking UPF consumption to thyroid function, particularly in childhood, remains limited, available evidence suggests that food processing may influence immunometabolic pathways involved in thyroid physiology and autoimmune disorders [25,27]. No study has yet established an association with paediatric thyroid outcomes.

### 3.2. Vegetarian and Vegan Diets: Potential Benefits and Nutritional Constraints for Thyroid Health

Vegetarian and vegan diets represent the most restrictive forms of plant-based diets and are characterised by the partial or complete exclusion of animal-derived foods. Their potential impact on thyroid health does not appear to depend on the absence of animal products itself, but rather on overall dietary quality, food variety, and the ability to provide adequate amounts of nutrients involved in thyroid hormone synthesis and metabolism.

Vegetarianism and veganism are frequently associated with more favourable cardiometabolic profiles, which may indirectly support endocrine homeostasis. However, evidence linking these diets to autoimmune thyroid diseases, including Graves’ disease (GD), is derived mainly from observational studies on adults and remains insufficient to establish causality [28].

The main concern regarding this diet is nutritional adequacy, particularly for micronutrients essential for thyroid function, including Iodine, selenium, iron, and zinc. Additionally, their bioavailability may be reduced by phytates present in plant foods [10,29]. Vitamin B12 and long-chain omega-3 fatty acids represent additional nutritional considerations [28,30].

These nutritional challenges are not inherent to vegetarian or vegan diets but are primarily associated with poor planning. Current evidence indicates that well-designed plant-based diets, characterised by adequate food variety and appropriate supplementation when required, support adequate nutritional status during childhood [4]. Compared with vegan diets, lacto-ovo-vegetarian and pescatarian patterns facilitate meeting nutritional requirements by including dairy products, eggs, or fish. Nevertheless, these diets also require appropriate planning to prevent potential micronutrient inadequacies [31].

## 4. Micronutrients, Vitamins and Potentially Goitrogenic Foods in Plant-Based Diets

Plant-based diets could interfere with thyroid hormone synthesis due to possible nutritional deficiencies in minerals and vitamins, and the consumption of goitrogenic foods and ultra-processed vegan alternatives.

### 4.1. Iodine

Iodine is a micronutrient crucial for the synthesis of the thyroid hormones, T3 and T4. Iodide (I-) is incorporated into the thyroid follicular cells through the iodide-sodium symporter (NIS) and into the tyrosine residues of thyroglobulin, giving rise to monoiodotyrosine (MIT) and diiodotyrosine (DIT), and then the thyroid hormones [32]. NIS is responsible for concentrating iodide in the thyroid gland and in other tissues, such as the mammary gland, placenta, and salivary and gastric mucosa [33]. The iodine not stored in these tissues is excreted in the urine and reflects iodine intake and nutritional status. The recommended daily intake of iodine changes according to age and the activity of the thyroid gland, which increases during puberty, pregnancy, and lactation. Table 1 summarises the recommended daily intake of iodine for the different ages of childhood [10,34].

In children, adults, and lactating women, iodine deficiency (ID) is defined as a median urinary iodine concentration (UIC) < 100 mcg/L (<150 mcg/L during pregnancy). Even if data on ID in the paediatric age are scarce, a German and Czech study documented that both vegan and vegetarian children had lower iodine intake when compared to omnivores [35,36]. A prolonged and marked deficiency in iodine compromises the synthesis of thyroid hormones and is considered the leading cause of hypothyroidism and nodular goitre worldwide. Variations in iodine intake, whether due to insufficient or excessive consumption, have been associated with thyroid dysfunction and a higher prevalence of autoimmune thyroid disorders. High iodine levels trigger a temporary inhibition of thyroid hormone production, a phenomenon known as the Wolff–Chaikoff effect. This response is followed by a reduction in the expression of the sodium-iodide symporter (NIS), allowing the thyroid gland to resume its normal function through the so-called escape from the Wolff–Chaikoff effect. When this adaptive mechanism fails, thyroid abnormalities such as hyperthyroidism, goitre, or hypothyroidism may develop [37].

Intake of iodine depends exclusively on diet and iodine supplements. Plant-based foods have a lower iodine content than animal-derived ones. The best iodine-containing foods include fish and other seafood. Eggs, milk, and their derivatives contain lower levels of iodine, depending on the use of iodine-fortified feed for farm animals or the use of fertilisers, country-specific policies on iodine fortification of food, and iodine concentrations in the soil and in fodder [38]. Thus, although ID is also found in a lacto-ovo vegetarian diet, the vegan diet contains very few foods with traces of iodine. In this type of diet, dried nori seaweed presents a major concentration of iodine (about 115.8 mcg of iodine/serving), even though its content of iodine is variable according to species and processing methods, and can even expose users to iodine excess, primarily if there is also an excessive use of iodine supplementation [39]. Other microelements and compounds, such as cassava, millet, and thiocyanates, could perturb iodine metabolism by competitively inhibiting iodine uptake by thyrocytes and facilitating the efflux of intrathyroidal iodine [40]. To reduce the risk of deficiency in vegetarian and vegan diets, the use of iodized salt is recommended according to national fortification programmes, together with iodine supplements providing 150 μg/day (225–250 μg/day during pregnancy) [28,39].

### 4.2. Selenium

Selenium is an essential micronutrient involved in antioxidant defence, immune function, and thyroid hormone metabolism. It is required for the activity of selenoproteins, including glutathione peroxidases (GPx) and iodothyronine deiodinases (DIOs) [38]. In the thyroid, GPx reduces hydrogen peroxide (H_2_O_2_), preserving thyroid peroxidase activity and protecting thyrocytes from oxidative damage [41]. In peripheral tissues, DIOs convert T4 into the active hormone T3 and reverse T3 (rT3).

Selenium deficiency has been associated with various thyroid disorders, including hypothyroidism, thyroid cancer, and autoimmune thyroid diseases [42].

Given the role of selenium in thyroid physiology, several studies have shown that selenium supplementation is associated with decreased circulating T4 and reverse T3 (rT3) concentrations in an iodine and selenium deficient population, while TSH and FT3 generally remain within the euthyroid range [43]. In addition, a systematic review and meta-analysis reported that selenium supplementation significantly reduced thyroid peroxidase antibody (TPOAb) levels in adult patients with autoimmune thyroiditis receiving levothyroxine (L-T4). However, the clinical relevance of this reduction remains uncertain [44]. Current international guidelines do not recommend routine selenium supplementation in all patients with autoimmune thyroiditis because evidence regarding clinical benefits remains inconsistent. Data on adequate selenium intake are reported in Table 1 [45]. Selenium is naturally present in foods in both inorganic and organic forms. The inorganic forms include selenate and selenite, while the organic forms are predominantly selenomethionine and selenocysteine [46]. Dietary selenium is obtained primarily from animal-derived foods, such as seafood, organ meats, eggs, milk, and dairy products, including low-fat and fat-free options.

**Table 1:** Recommended Dietary Reference Intakes for nutrients relevant to thyroid physiology in paediatric age.

TRACE Elements	Recommended Dietary Allowance (RDAs) and Adequate Intake (AI)	Role in Thyroid Health
Iodine	Birth-6 months: 110 mcg/day (AI) 7 to 12 months: 130 mcg/day (AI) 1 year to school age: 90 mcg/day Puberty (9–13 years): 120 mcg/day Pregnancy/lactation: 250 mcg/day	Essential constituent of MIT, DIT and thyroid hormones (T3 and T4)
Selenium	Birth-6 months: 15 mcg/day (AI) 7 to 12 months: 20 mcg/day (AI) 1–3 years: 20 mcg/day 4–8 years: 30 mcg/day 9–13 years: 40 mcg/day 14–18 years and Adults: 55 mcg/day	Required for selenoprotein, as DIOs, GPx, and TrxR implicated in thyroid function and antioxidant defence
Iron	Birth-6 months: 0.27 mg/day (AI) 7 to 12 months: 11 mg/day 1 to 3 years: 7 mg/day 4 to 10 years: 8–10 mg/day 9 to 13 years: 8 mg/day males 14–18 years: 11 mg/day females 14–18 years: 15 mg/day	Iron permits the function of thyroid peroxidase, a heme-containing enzyme, and catalyses the first two stages of thyroid hormone synthesis.
Zinc	0–6 months: 2 mg/day (AI) 7–12 months: 3 mg/day 1–3 years: 3 mg/day 4–8 years: 5 mg/day 9–13 years: 8 mg/day males 14–18 years: 11 mg/day females 14–18 years: 9 mg/day	Zinc influences the deiodinase activity, regulates the secretion of TRH and TSH

Values are US Dietary Reference Intakes established by the Food and Nutrition Board of the National Academies [45]. AI, Adequate Intake; DIOs, iodothyronine deiodinases; DIT, diiodotyrosine; GPx, glutathione peroxidases; MIT, monoiodotyrosine; RDAs, Recommended Dietary Allowance; T3, triiodothyronine; T4, thyroxine; TRH, thyrotropin-releasing hormone; TrxR, thioredoxin reductases; TSH, thyroid-stimulating hormone. AI is used when evidence is insufficient to establish an RDAs. These values apply to apparently healthy individuals and should not be interpreted as therapeutic supplementation doses.

Nevertheless, several plant-based foods also contribute to selenium intake, including Brazil nuts, sunflower seeds, cereals, fruits, and vegetables such as onions, green peas, peppers, grapes, and potatoes. The selenium content of plant foods can vary substantially because soil selenium concentrations and other environmental conditions strongly influence it [47].

### 4.3. Iron

Iron plays a crucial role in numerous metabolic processes within the human body, including the production of thyroid hormones. Thyroid peroxidase (TPO), a heme-containing enzyme, requires iron for its activity and is responsible for catalysing the first two stages of thyroid hormone synthesis. Iron is also an essential component of the prosthetic group of catalases, important antioxidant enzymes that protect cells by promoting the breakdown and detoxification of hydrogen peroxide [48]. Thus, iron deficiency may impair thyroid function, and hypothyroidism may coexist with other clinical conditions, such as anaemia, due to chronic and persistent iron deficiency. Autoimmune thyroid disorders are associated with autoimmune gastritis, coeliac disease, and inflammatory bowel disease. These gastrointestinal conditions may lead to malabsorption of iron and other micronutrients, including vitamin B12, folate, and iodine, with potential consequences for thyroid function. [49,50,51].

Although evidence in paediatric populations remains limited, combined iron and iodine supplementation has been associated with a significantly smaller thyroid volume than iodine supplementation alone, suggesting that preventing iron deficiency enhances the efficacy of iodine prophylaxis. Deficiencies in iron, selenium, and zinc may exacerbate the detrimental effects of ID on thyroid function while impairing the antioxidant defence mechanisms of thyrocytes [52]. Depending on iron nutritional status, the recommended daily iron intake varies by age and sex, increasing during puberty. Table 1 summarises the daily intake of iron [45,53].

Dietary iron is available in two main forms: heme iron and non-heme iron. Heme iron originates from haemoglobin and myoglobin and is found exclusively in foods of animal origin, such as red meat, poultry, fish, liver, shellfish, oysters, and clams. Non-heme iron is predominantly present in plant-based foods, including legumes, beans, lentils, whole grains, nuts, seeds, and green leafy vegetables, and the iron in this form is commonly added to fortify food products [54]. Although the typical daily intake of iron ranges from 10 to 15 mg, only about 1–2 mg is absorbed by the gastrointestinal tract. The efficiency of absorption largely depends on the chemical form of iron. Heme iron is generally more bioavailable than non-heme iron and the absorption is less influence by other dietary components.

In contrast, the absorption of non-heme iron is highly affected by diet [54,55]. Vitamin C markedly enhances absorption whereas compounds that inhibit absorption include phytates in whole grains, legumes, nuts, and seeds, tannins and polyphenols found in tea, calcium-rich foods, oxalates present, for example, in chocolate and rhubarb, and soy-based products. Therefore, the iron intake of people following narrow plant-based diets, with limited or no animal-derived foods, is heavily or entirely dependent on non-heme iron [56,57] which can lead to an elevated risk of iron deficiency, especially during life stages with higher iron requirements, such as childhood, puberty, and pregnancy.

### 4.4. Zinc

Zinc is a vital trace element that plays an important role in thyroid hormone metabolism and endocrine regulation. It participates in the synthesis and activation of thyroid hormones by influencing deiodinase activity, regulating the secretion of thyrotropin-releasing hormone (TRH) and thyroid-stimulating hormone (TSH), and modulating transcription factors involved in thyroid hormone production. As a result, nutritional status of zinc has been associated with variations in circulating levels of TSH, T3 and T4 [58].

Adequate zinc intake is essential during childhood and adolescence. The average dietary zinc intake has been estimated at 7–10 mg/day among children and adolescents. These figures are generally in line with current dietary recommendations, which range from approximately 2–3 mg/day during childhood to 8–11 mg/day during adolescence, depending on age and sex. Maintaining an adequate zinc intake is particularly important during periods of rapid growth and development, given its role in immune function, cell proliferation, and endocrine regulation [59,60].

From a nutritional perspective, the primary dietary sources of zinc are meat, poultry, seafood, and other animal-derived foods, which provide highly bioavailable zinc. Plant-based diets can also supply adequate amounts of zinc through cereals, legumes, nuts, and seeds; however, zinc bioavailability is often reduced by the presence of phytates and, to a lesser extent, oxalates, which form insoluble complexes and limit intestinal absorption [61]. Consequently, individuals following vegetarian or vegan dietary patterns may require a proportionally higher dietary zinc intake to compensate for reduced bioavailability. Various food-processing methods can improve the bioavailability of zinc from plant-based foods. Techniques such as soaking, germination, milling, and fermentation help lower phytate concentrations by activating endogenous phytases, thereby reducing one of the main inhibitors of zinc absorption. Certain dietary components, including organic acids and sulphur-containing amino acids, can promote zinc uptake and enhance its absorption in the gastrointestinal tract [62]. Therefore, careful dietary planning is recommended for individuals adhering to strict plant-based diets to ensure adequate zinc intake and support optimal thyroid and overall metabolic health. Table 1 summarises also the recommended daily intake of iron.

### 4.5. Vitamins

Vitamin D may likewise influence thyroid immune homeostasis through its immunomodulatory properties. In addition to its role in calcium and bone metabolism, vitamin D regulates both innate and adaptive immune responses through the vitamin D receptor, which is expressed in numerous immune cells and in thyroid tissue [63]. Genetic studies have identified associations between vitamin D receptor polymorphisms and susceptibility to autoimmune disorders, including GD, Hashimoto’s thyroiditis (HT), and postpartum thyroiditis (PPT) [64]. Observational studies and meta-analyses report lower vitamin D levels in patients with HT and an association between vitamin D deficiency, thyroid autoimmunity, and higher autoantibody levels. Whether deficiency contributes to disease development or reflects related inflammatory and metabolic changes remains unclear, especially given the high prevalence of deficiency in the general population, including in children and adolescents. [65]. From a nutritional perspective, dietary sources of vitamin D are relatively limited and include oily fish, cod liver oil, egg yolk, and dairy products, making adequate intake potentially challenging in individuals following strict plant-based diets [66]. Therefore, along with vitamin B12, careful assessment and management of vitamin D status may be an important component of nutritional care in people on plant-based diets, particularly among individuals with autoimmune thyroid disorders.

Other vitamins may also be deficient in plant-based diets, including vitamin B12 and vitamin A, which play roles in immune regulation and cellular methylation [67].

### 4.6. Soy-Based Products and Potentially Goitrogenic Foods

Soy-based foods are among the most controversial components of plant-based diets in relation to thyroid health. Concerns primarily arise from the content of isoflavones, particularly genistein and daidzein, which have been shown to interfere in vitro with thyroid hormone synthesis through inhibition of TPO, the enzyme responsible for iodine organification and coupling reactions. Soy isoflavones can reduce TPO activity and impair thyroid hormone biosynthesis, providing a mechanistic basis for their classification as goitrogenic compounds [68]. However, the clinical relevance of these findings depends on the nutritional and physiological context.

Iodine status is key in determining whether soy consumption has clinically significant effects on thyroid function. Soy is not intrinsically harmful to the thyroid but may become problematic in the context of inadequate iodine intake. In individuals with adequate iodine intake, the consumption of soy foods appears to have minimal or no influence on circulating thyroid hormone levels or TSH concentrations. Insufficient iodine intake, however, can enhance the antithyroid properties of soy, increasing the likelihood of goitre development and thyroid dysfunction. This interaction is particularly important during childhood. Before the widespread adoption of iodine fortification, cases of goitre and hypothyroidism were documented in infants fed with soy-based formulas; these alterations frequently improved following iodine supplementation or changes in dietary intake [69].

Plant-based diets also often include cruciferous vegetables such as broccoli, cauliflower, cabbage, kale, and Brussels sprouts, which are traditionally classified as goitrogenic. These vegetables contain glucosinolates and their metabolites, including thiocyanates and isothiocyanates, which can competitively inhibit iodine uptake in the thyroid gland through interference with NIS. However, a comprehensive systematic review concluded that normal consumption of Brassica vegetables does not adversely affect thyroid function in humans and that potential adverse effects are largely restricted to situations combining excessive intake with ID [70]. Similar conclusions have emerged from previous reviews, indicating that concerns regarding cruciferous vegetables have likely been overstated [71].

Overall, the thyroidal effects of soy products and other goitrogenic foods are context-dependent rather than intrinsically harmful. In paediatric populations, where physiological vulnerability is greater, the key determinant appears to be adequate iodine intake. Thus, within a well-planned plant-based diet that meets iodine requirements, moderate consumption of soy foods and cruciferous vegetables does not appear to pose a significant risk to thyroid health. In contrast, caution may be warranted in children with ID or pre-existing thyroid disorders.

## 5. Paediatric Implications

### 5.1. Thyroid Hormones During Growth and Neurodevelopment

Thyroid hormones exert a different biological role during development and the paediatric age than in adulthood. [72]. During foetal life and throughout childhood, T3 and T4 act as developmental signals required for the maturation of multiple organs, particularly the central nervous system. They regulate highly time-dependent processes, including neuronal proliferation, migration and differentiation, synaptogenesis, dendritic arborization, and myelination [73,74]. Accordingly, thyroid function assumes a unique biological importance during childhood. Mild or transient thyroid dysfunction in adults is often reversible after restoration of euthyroidism; in contrast, reduced thyroid hormone availability during development may disrupt biological processes that are only partially reversible, leading to persistent alterations in brain architecture and neurocognitive function [74,75].

This developmental role explains why childhood is a particularly vulnerable period for nutritional inadequacies that influence thyroid hormone synthesis, metabolism, and action. In paediatric populations, even relatively modest nutritional inadequacies may be more clinically relevant than in adults because they occur during phases of rapid growth and organ maturation, when endocrine disturbances may lead to long-term developmental consequences [73,74].

### 5.2. Early-Life Vulnerability to Nutritional Inadequacy in Plant-Based Diets

Early life represents a period of nutritional vulnerability, characterised by increased requirements for essential nutrients to support somatic growth, organ maturation and the development of endocrine and metabolic systems. Even modest variations in the availability of essential nutrients may have greater biological consequences than in adulthood, particularly when occurring during periods of rapid development [76,77,78]. Nutritional adequacy depends on maternal nutritional status during pregnancy and breastfeeding, dietary intake, as well as on complementary foods.

Iodine is an essential substrate for thyroid hormone synthesis. Iodine requirements rise during pregnancy to support maternal thyroid hormone production and the progressive maturation of foetal and neonatal thyroid function [10,34]. During pregnancy, thyroid hormone activity relies largely on maternal thyroid function, making maternal iodine status critical during the earliest stages of brain development [73,79].

In breastfed infants, iodine intake depends almost exclusively on the iodine content of human milk, which directly reflects maternal iodine intake and status. By contrast, infant formulas are subject to compositional regulations that ensure adequate iodine content [80,81]. However, similar requirements are not uniformly applied to complementary foods for early childhood, and some commercially available products provide insufficient iodine to meet recommended intakes [10,82,83].

Children following appropriately planned vegetarian and vegan diets can meet nutritional requirements during growth but the exclusion of major dietary sources of iodine, such as seafood and dairy products, may increase the risk of suboptimal iodine intake when fortified foods or alternative iodine sources are not adequately included [35,36].

The clinical relevance of early-life ID lies primarily in its effects on brain development. Reduced thyroid hormone synthesis in utero, the neonatal period, or early childhood may impair neurodevelopmental processes, including neuronal proliferation and migration, myelination, and synaptic maturation [74,84]. While severe ID represents a well-established and preventable cause of impaired neurodevelopment, even mild-to-moderate iodine insufficiency in utero and during childhood has been associated with poorer neurocognitive outcomes, including alterations in cognitive performance, language development, learning abilities, and executive functions [84,85,86].

### 5.3. Growth-Related Nutritional Demands

Elevated physiological needs during childhood and adolescence make these stages especially susceptible to nutritional imbalances. Rapid growth, skeletal development, brain maturation, and hormone regulation occur concurrently, raising the demands for energy, protein, and vital micronutrients. As a result, insufficient intake of nutrients essential for correct thyroid functioning are more biologically significant in these periods than in adulthood [4,87].

As well as iodine, particular attention should be paid to iron, zinc, and selenium, which contribute to thyroid hormone synthesis, metabolism, and biological action through distinct mechanisms. In insufficiently planned plant-based diets, intake of these nutrients may be suboptimal [10,29]. Nutritional needs differ across developmental stages: in early childhood, nutrients are crucial for rapid brain and endocrine system development; during adolescence, growth spurts, muscle development, and puberty increase the need for key micronutrients like iron and zinc. Deficiencies in these nutrients can disrupt thyroid balance and development [29,74].

Iron is particularly relevant because it is required for TPO activity, in the neurodevelopmental processes, including myelination, neuronal energy metabolism, and neurotransmitter synthesis. Zinc supports cellular proliferation, linear growth, and pubertal maturation, whereas selenium permits the activity of deiodinases and other selenoproteins that provide antioxidant protection for the thyroid gland. Reduced availability of these micronutrients may affect both thyroid homeostasis and normal growth and development [14,29,58,88,89,90,91].

Current evidence indicates that well-planned plant-based diets can support normal growth throughout childhood and adolescence. Nevertheless, studies have reported differences in the intake or status of potentially critical micronutrients, particularly iron, zinc, and selenium, among vegetarian and vegan children compared with omnivorous peers, underscoring the importance of careful dietary planning and individualised nutritional monitoring throughout growth [4,87,92].

These paediatric findings concern nutrient intake and status and do not, by themselves, demonstrate associated thyroid dysfunction.

### 5.4. Puberty as a Critical Developmental Period

Adolescence represents a potential phase of endocrine vulnerability. During this period, thyroid hormones contribute to the regulation of linear growth, skeletal maturation, and the functional integration of endocrine pathways involved in development, particularly the growth hormone (GH)/insulin-like growth factor-1 (IGF-1) axis and sex steroid signalling [93,94].

During puberty, increased GH and IGF-1 secretion, together with activation of the hypothalamic–pituitary–gonadal axis, drive the pubertal growth spurt and skeletal maturation. Thyroid hormones play a permissive role in these processes by promoting growth plate activity and supporting the normal acquisition of bone mass. Consequently, alterations in thyroid function during this period may lead to reduced growth velocity, delayed skeletal maturation, and impaired pubertal progression [93,94].

Puberty is also characterised by increased nutritional demands to sustain rapid growth and reproductive maturation. In adolescents on plant-based diets, careful dietary planning is essential [10,29]. Iron deserves particular attention because its requirements increase substantially during adolescence, especially after menarche. Given the lower bioavailability of non-heme iron from plant foods, inadequate intake may further compromise TPO activity and the physiological processes supporting growth during this stage [54,88].

Clinical management of adolescents on plant-based diets should emphasise overall dietary adequacy and appropriate nutritional monitoring [4].

### 5.5. Children with Pre-Existing Thyroid Disease

Children and adolescents with pre-existing thyroid disorders constitute a population for whom nutritional adequacy is particularly important. Chronic autoimmune thyroiditis is the most common cause of acquired hypothyroidism during childhood and adolescence and is characterised by a progressive reduction in the thyroid functional reserve [95].

Current evidence does not indicate that plant-based diets intrinsically worsen thyroid autoimmunity in paediatric patients with thyroid disease, but diet quality and micronutrient content should be monitored. As micronutrients are involved in essential processes related to thyroid hormone synthesis, metabolism, and biological activity, reduced availability of these nutrients may contribute to greater functional instability when thyroid reserve is already compromised [10,29,89]. Children with autoimmune thyroiditis or other thyroid disorders following a plant-based diet should be regularly checked for nutritional status growth and pubertal development [4,87].

## 6. Early-Life Immune Programming and Thyroid Health

Beyond the direct effects of micronutrients on thyroid physiology, nutrition during early life may influence thyroid health through more complex mechanisms, including immune programming and modulation of the gut microbiota. According to the Developmental Origins of Health and Disease (DOHaD) paradigm, nutritional factors during pregnancy and the first years of life can induce persistent changes in endocrine, metabolic, and immune development and modulate disease susceptibility later in life [96,97].

One of the principal mediators of these processes is the gut microbiota, whose development is shaped in early life by mode of delivery, breastfeeding, antibiotic exposure, complementary feeding, and overall diet. During infancy, the microbiota supports immune system maturation, maintains immune tolerance, and regulates systemic inflammatory responses [98,99,100].

In plant-based diets, effects on the gut microbiota appear to depend primarily on the overall quality of food. A diet rich in minimally processed plant foods may promote the growth of bacteria that produce short-chain fatty acids (SCFAs), including acetate, propionate, and butyrate. These microbial metabolites help maintain intestinal barrier integrity and regulate immune pathways, including modulation of regulatory T-cell responses [17,18].

High intakes of UPFs, even plant-based UPFs, has been linked to reduced microbial diversity, altered SCFA production, and increased pro-inflammatory signalling. These findings suggest that the immunometabolic effects of plant-based diets are largely determined by dietary quality rather than by the exclusion of animal products [25,101].

These mechanisms have gained increasing attention in autoimmune thyroid diseases. The existence of a microbiota–thyroid axis has been proposed through which gut microbial composition and activity may influence thyroid hormone metabolism and immune responses to thyroid antigens. Additionally, the gut microbiota may influence the absorption and processing of nutrients essential for thyroid health, such as iodine, selenium, iron, and zinc, and could impact peripheral thyroid hormone metabolism and enterohepatic circulation [102,103].

Alterations in gut microbiota composition have been reported in individuals with autoimmune thyroid diseases but current evidence remains predominantly observational and does not establish causal relationships between dysbiosis and thyroid autoimmunity [104,105]. The thyroid-specific evidence derives predominantly from adult observational studies, while mechanistic support also comes from animal and in vitro studies; application to children therefore remains an indirect inference.

**Figure 1:**
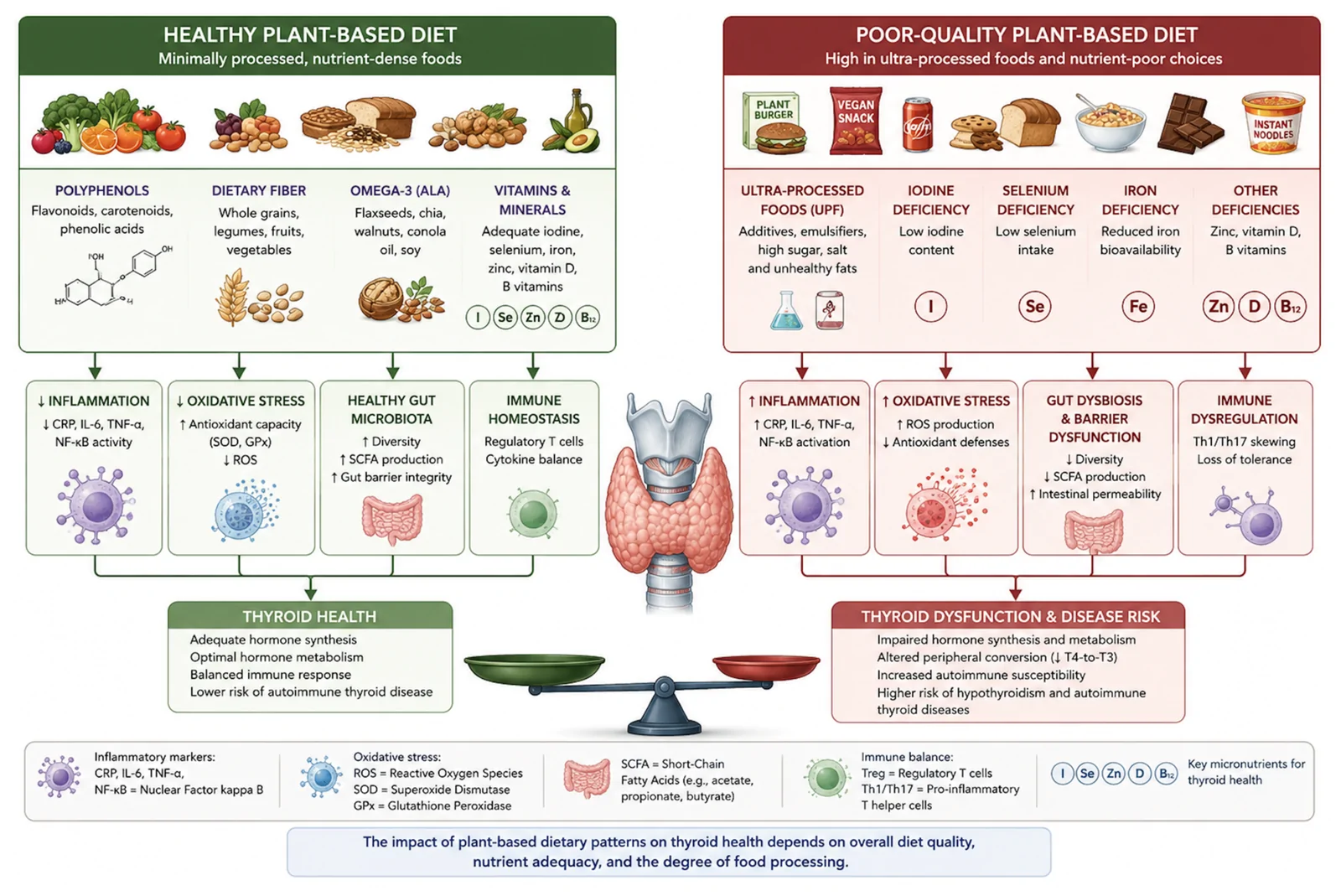
Proposed pathways linking the quality of plant-based diets to thyroid health. How the quality of plant-based diets may influence thyroid homeostasis through micronutrient availability and oxidative, inflammatory, immune, and gut microbial pathways. The proposed mechanisms are largely based on observational and mechanistic evidence and do not establish causality, particularly in paediatric populations. Created with BioRender.com. Abbreviations: ALA, α-linolenic acid; CRP, C-reactive protein; GPx, glutathione peroxidase; IL-6, interleukin-6; NF-κB, nuclear factor kappa B; ROS, reactive oxygen species; SCFAs, short-chain fatty acids; SOD, superoxide dismutase; T3, triiodothyronine; T4, thyroxine; Th1, T-helper 1 cells; Th17, T-helper 17 cells; TNF-α, tumour necrosis factor alpha; Treg, regulatory T cells; UPFs, ultra-processed foods.

Available evidence suggests that the potential influence of plant-based diets on thyroid health during childhood may extend beyond micronutrient availability, encompassing broader mechanisms that involve modulation of the gut microbiota and immune programming during critical developmental periods (Figure 1). However, the clinical relevance of these mechanisms has not been fully elucidated and requires prospective studies conducted specifically in paediatric populations [106,107].

## 7. Conclusions

Available evidence does not show that well-planned plant-based diets are intrinsically harmful to thyroid health in children. However, direct paediatric evidence based on thyroid outcomes is limited. Iodine inadequacy is the most clearly documented nutritional concern, while evidence linking selenium, iron, zinc, vitamin D, food processing, inflammation and the gut microbiota to paediatric thyroid dysfunction is largely indirect.

Since childhood and adolescence are critical periods of growth and hormonal development, clinical efforts should focus on proper dietary planning and personalised monitoring of nutritional status, growth, and key nutrient intake in children following plant-based diets. At routine paediatric visits, assessment should include dietary iodine sources, use of fortified foods and supplements, and growth trajectory. Thyroid function testing and subsequent monitoring should be guided by nutritional risk and clinical findings. In children with pre-existing thyroid disease, dietary assessment should be integrated into established endocrine follow-up, with particular attention to iodine adequacy and avoidance of excessive supplementation. Future paediatric research should move beyond an focusing on individual nutrients and consider the complex interplay among food quality, food processing, micronutrients, the gut microbiota, and immune regulation in relation to thyroid function and autoimmunity.

## Data Availability

Data sharing does not apply to this article as no new data were created or analysed in this study.

## Abbreviations

The following abbreviations are used in this manuscript:

AI	Adequate intake
ALA	α-Linolenic acid
CRP	C-reactive protein
DIOs	Iodothyronine deiodinases
DIT	Diiodotyrosine
DOHaD	Developmental Origins of Health and Disease
FT3	Free triiodothyronine
GD	Graves’ disease
GH	Growth hormone
GPx	Glutathione peroxidase(s)
HT	Hashimoto’s thyroiditis
ID	Iodine deficiency
IGF-1	Insulin-like growth factor-1
IL-6	Interleukin-6
L-T4	Levothyroxine
MAPK	Mitogen-activated protein kinase
MD	Mediterranean diet
MEDLINE	Medical Literature Analysis and Retrieval System Online
MIT	Monoiodotyrosine
NF-κB	Nuclear factor kappa-light-chain-enhancer of activated B cells
NIS	Sodium–iodide symporter
PPT	Postpartum thyroiditis
RDAs	Recommended dietary allowances
ROS	Reactive oxygen species
rT3	Reverse triiodothyronine
SCFA(s)	Short-chain fatty acid(s)
SOD	Superoxide dismutase
T3	Triiodothyronine
T4	Thyroxine
Th1	T-helper 1 cells
Th17	T-helper 17 cells
TNF-α	Tumour necrosis factor alpha
TPO	Thyroid peroxidase
TPOAb	Thyroid peroxidase antibodies
Treg	Regulatory T cells
TRH	Thyrotropin-releasing hormone
TrxR	Thioredoxin reductase(s)
TSH	Thyroid-stimulating hormone
UIC	Urinary iodine concentration
UPF(s)	Ultra-processed food(s)
ω-3 PUFAs	Omega-3 polyunsaturated fatty acids
